# Validation and comparison of clinical prediction rules for invasive candidiasis in intensive care unit patients: a matched case-control study

**DOI:** 10.1186/cc10366

**Published:** 2011-08-09

**Authors:** Elizabeth D Hermsen, Michelle K Zapapas, Melissa Maiefski, Mark E Rupp, Alison G Freifeld, Andre C Kalil

**Affiliations:** 1 Department of Pharmaceutical & Nutrition Care, The Nebraska Medical Center, 981090 Nebraska Medical Center, Omaha, NE 68198-1090, USA; 2 Section of Infectious Diseases, Department of Internal Medicine, College of Medicine, University of Nebraska Medical Center, 985400 Nebraska Medical Center, Omaha, NE 68198-5400, USA; 3 Department of Pharmacy Practice, College of Pharmacy, University of Nebraska Medical Center, 986045 Nebraska Medical Center, Omaha, NE 68198-6045, USA

**Keywords:** candidiasis, clinical prediction rules, prophylaxis

## Abstract

**Introduction:**

Due to the increasing prevalence and severity of invasive candidiasis, investigators have developed clinical prediction rules to identify patients who may benefit from antifungal prophylaxis or early empiric therapy. The aims of this study were to validate and compare the Paphitou and Ostrosky-Zeichner clinical prediction rules in ICU patients in a 689-bed academic medical center.

**Methods:**

We conducted a retrospective matched case-control study from May 2003 to June 2008 to evaluate the sensitivity, specificity, positive predictive value (PPV) and negative predictive value (NPV) of each rule. Cases included adults with ICU stays of at least four days and invasive candidiasis matched to three controls by age, gender and ICU admission date. The clinical prediction rules were applied to cases and controls via retrospective chart review to evaluate the success of the rules in predicting invasive candidiasis. Paphitou's rule included diabetes, total parenteral nutrition (TPN) and dialysis with or without antibiotics. Ostrosky-Zeichner's rule included antibiotics or central venous catheter plus at least two of the following: surgery, immunosuppression, TPN, dialysis, corticosteroids and pancreatitis. Conditional logistic regression was performed to evaluate the rules. Discriminative power was evaluated by area under the receiver operating characteristic curve (AUC ROC).

**Results:**

A total of 352 patients were included (88 cases and 264 controls). The incidence of invasive candidiasis among adults with an ICU stay of at least four days was 2.3%. The prediction rules performed similarly, exhibiting low PPVs (0.041 to 0.054), high NPVs (0.983 to 0.990) and AUC ROCs (0.649 to 0.705). A new prediction rule (Nebraska Medical Center rule) was developed with PPVs, NPVs and AUC ROCs of 0.047, 0.994 and 0.770, respectively.

**Conclusions:**

Based on low PPVs and high NPVs, the rules are most useful for identifying patients who are not likely to develop invasive candidiasis, potentially preventing unnecessary antifungal use, optimizing patient ICU care and facilitating the design of forthcoming antifungal clinical trials.

## Introduction

The prevalence of nosocomial invasive candidiasis has increased dramatically since the 1990s, with *Candida *spp. being the fourth most common cause of bloodstream infections in the United States, accounting for 9% of such infections, over half of which occur among ICU patients [1,2]. Some risk factors commonly associated with invasive candidiasis include prior surgery, acute renal failure, receipt of total parenteral nutrition (TPN) and presence of a central venous catheter (CVC) [3]. The clinical and economic burden of invasive candidiasis is significant, as these infections are associated with increased mortality, longer hospital stay and higher cost [4]. Crude mortality in patients with candidemia is approximately 40%, with the highest mortality among those infected with *C. krusei *(approaching 60%) and the lowest among those infected with *C. parapsilosis *(approaching 30%) [2]. Delayed initiation of appropriate antifungal therapy for candidemia has been associated with increased mortality [5-7].

In an effort to minimize the detrimental effects of nosocomial candidiasis and delayed appropriate therapy, prophylactic antifungal therapy has been utilized with varied results [8]. Some studies of ICU patients have shown a beneficial effect of antifungal prophylaxis [9-11], but others have not [12]. Two meta-analyses have shown conflicting results, one showing decreased mortality with antifungal prophylaxis [13] and the other showing no survival advantage [14]. Nevertheless, universal antifungal prophylaxis is not clinically or fiscally responsible, as many patients are at low risk for developing infection and inappropriate use of antifungals can promote resistance and increase costs. Therefore, a prediction rule for identifying patients most likely to benefit from prophylaxis would be advantageous.

In 2005, Paphitou and colleagues [15] published a prediction rule for invasive candidiasis in ICU patients that incorporated risk factors of hemodialysis, TPN, diabetes mellitus and broad-spectrum antibiotics. In 2007, Ostrosky-Zeichner and colleagues [16] sought to improve upon the previously published rule. They found that a CVC or broad-spectrum antibiotics in combination with at least two minor risk factors (pancreatitis, major surgery, TPN, immunosuppressants, corticosteroids or dialysis) was predictive of invasive candidiasis.

Limited data regarding external validation of the Ostrosky-Zeichner rule exist [17,18], and no published evidence indicates external validation of the Paphitou rule. The aim of our study was to evaluate the Paphitou [15] and Ostrosky-Zeichner [16] clinical prediction rules and retrospectively validate them in ICU patients at our institution.

## Materials and methods

### Population

This study was a retrospective, matched case-control study conducted at The Nebraska Medical Center, a 689-bed academic medical center. Cases and controls were eligible for inclusion if they had an ICU stay of four days or longer from May 2003 through June 2008 and were at least 18 years old. Exclusion criteria included invasive candidiasis or receipt of systemic antifungals prior to day 4 of the ICU stay. Inclusion and exclusion criteria matched those of the original studies [15,16]. Patients who developed invasive candidiasis from day 4 of the ICU stay through seven days after ICU discharge were identified on the basis of clinical microbiology data and served as the case cohort. Cases were matched at a 1:3 ratio with patients who did not develop invasive candidiasis (controls), according to negative culture results, based on age, gender and ICU admission date. The overall incidence of invasive candidiasis was determined by the entire ICU cohort of all patients at least 18 years old with an ICU stay of at least 4 days admitted during the study period (not limited to cases and controls). The clinical prediction rules were applied to cases and controls via retrospective chart review to evaluate the success of the rules in predicting invasive candidiasis. The study was approved by our medical center's Institutional Review Board with a complete waiver of informed consent.

### Data collection

Invasive candidiasis risk factor data were collected starting from seven days prior to ICU admission (D-7) through the third day of ICU stay (D3) and included presence of a CVC, TPN, hemodialysis, major surgery, pancreatitis, mechanical ventilation, Acute Physiology and Chronic Health Evaluation (APACHE) II score (calculated on D1), history of invasive candidiasis, broad-spectrum antibiotics, systemic corticosteroids and immunosuppressants. Outcome data were collected from day 4 of the ICU stay through 7 days after ICU discharge and included cultures positive for *Candida *spp. from blood, peritoneal fluid or another sterile site. Urine, sputum or bronchial washing positive for *Candida *spp. were not considered invasive candidiasis. The diagnosis of invasive candidiasis was made based on the revised consensus definitions of invasive fungal infections developed by the European Organization for Research and Treatment of Cancer/Invasive Fungal Infections Cooperative Group and the National Institute of Allergy and Infectious Disease Mycoses Study Group [19]. More specifically, only those with proven invasive candidiasis, as demonstrated by recovery of yeast from a sample obtained from a normally sterile site, were included as cases. Major surgery was defined as a procedure utilizing general anesthesia. Pancreatitis was identified using International Classification of Diseases, Ninth Revision, code 577.0 (acute pancreatitis) as a primary or secondary discharge diagnosis. From the electronic medical records, we collected data regarding administration of medications and TPN; culture, chemistry and hematology profiles; history of invasive candidiasis; and diabetes. Data regarding mechanical ventilation, vital signs, surgeries, ICU discharge date and CVC were collected from the paper medical records. Discharge disposition and cost of stay data were collected from the institution's financial database.

### Clinical prediction rules

The Paphitou rule included receipt of TPN (D-7 to D0), hemodialysis (D1 to S3) or a history of diabetes mellitus with or without broad-spectrum antibiotics (D-7 to D3). For purposes of this study, two Paphitou rules were evaluated: one without broad spectrum antibiotics and one with broad spectrum antibiotics (Table 1). The Ostrosky-Zeichner rule included one of two major risk factors (CVC (D1 to D3), receipt of broad-spectrum antibiotics (D1 to D3)) and at least two minor risk factors (TPN (D1 to D3), dialysis (D1 to D3), major surgery (D-7 to D0), pancreatitis (D-7 to D0), corticosteroids (D-7 to D3) or immunosuppressants (D-7 to D0)). For purposes of this study, two Ostrosky-Zeichner rules were evaluated: one with broad-spectrum antibiotics and one with a CVC (Table 1).

**Table 1:** Paphitou and Ostrosky-Zeichner clinical prediction rules^a^

Rule	Criteria		
Paphitou 1	≥1: TPN (D-7 to D0), hemodialysis (D1 to D3), diabetes		
Paphitou 2	≥1: TPN (D-7 to D0), hemodialysis (D1 to D3), diabetes, broad-spectrum antibiotics (D-7 to D3)		
Ostrosky-Zeichner 1	Central venous catheter (D1 to D3)	and ≥2	TPN (D1 to D3), hemodialysis (D1 to D3), major surgery (D-7 to D0), pancreatitis (D7 to D0), corticosteroids (D-7 to D3), immunosuppressants (D-7 to D0)
Ostrosky-Zeichner 2	Broad spectrum antibiotics (D1 to D3)		

^a^TPN, total parenteral nutrition; D, day of intensive care unit stay. Adapted from [15,16].

A breakpoint value was calculated for each clinical prediction rule based on the final regression equation for each specific clinical prediction rule. The breakpoint values provide a practical means by which to interpret the results of each equation after they are applied to an individual patient. For example, if the resultant value is below the breakpoint of the clinical prediction rule, then according to the clinical prediction rule, antifungal therapy will not be recommended, and vice versa.

### Statistical analysis

Baseline categorical variables were analyzed using the χ^2 ^test, and continuous variables were analyzed using Student's *t*-test. Univariate regression was performed to select risk factors to be entered into the multivariable model. Risk factors with *P *< 0.20 in the univariate regression were selected for the backward elimination stepwise conditional logistic regression model. Each case and its three controls, matched on age, gender and ICU admission date, were used as a stratum in the conditional logistic regression analysis [20]. The final model included only variables with *P *< 0.05. These variables from the final model were used to construct the equations used for the clinical prediction rules. An institution-specific clinical prediction rule, named the Nebraska Medical Center rule (NMC rule), was developed based on the institutional risk factors demonstrating statistical significance following the conditional logistic regression modeling. The prediction rules were applied to our patient population, and the primary outcomes were sensitivity, specificity, positive predictive value (PPV) and negative predictive value (NPV) of each of the clinical prediction rules for invasive candidiasis. A receiver operating characteristic (ROC) curve was generated for each rule, and the area under the ROC (AUC ROC) curve was calculated to determine their discriminative power. SPSS version 17.0 software (SPSS Inc., Chicago, IL, USA) was used for all analyses.

## Results

### Patient characteristics

From May 2003 through June 2008, 100 cases met our inclusion criteria. Four of these cases developed invasive candidiasis prior to day 4 and were excluded. Eight patients received systemic antifungals prior to day 4 and were excluded. Eighty-eight cases were included and matched to controls at a 1:3 ratio, yielding a total of 352 patients. Of these patients, 59% were male, 75.9% were Caucasian, 9.9% were African-American and the remaining 14.2% were Hispanic or of other ethnic origin. The mean age of the patient population was 60.3 years (range, 21 to 96 years). The overall incidence of invasive candidiasis during the study period for all patients at least 18 years old with an ICU stay of at least 4 days was 23.3 cases per 1,000 ICU patient-years.

### Risk factors

Table 2 shows the results of conditional logistic regression for the occurrence of risk factors in cases versus matched controls. The following risk factors demonstrated statistically significant differences: any broad-spectrum antibiotic use, CVC (D1 to D3), abdominal surgery (D-7 to D3), immunosuppressants (D-7 to D0), TPN (D1 to D3) and mean pre-ICU length of stay. These risk factors were used to derive the institution-specific rule (NMC rule), except for systemic corticosteroid use, which was used instead of immunosuppressants because corticosteroid use was more common, is likely to be more applicable to other populations and showed a trend toward statistical significance.

**Table 2:** Presence of risk factors in cases versus controls^a^

Risk factor (days of ICU stay)	All (*n *= 352) *n *(%)	Cases (*n *= 88) *n *(%)	Controls (*n *= 264) *n *(%)	*P *value	OR (95% CI)
Broad-spectrum antibiotics (-7 to 0)	80 (22.7)	30 (34.1)	50 (18.9)	0.005	2.21 (1.29 to 3.79)
Broad-spectrum antibiotics (1 to 3)	296 (84.1)	85 (96.6)	211 (79.9)	< 0.001	7.12 (2.17 to 23.4)
Broad-spectrum antibiotics (-7 to 3)	298 (84.7)	85 (96.6)	213 (80.7)	< 0.001	6.74 (2.06 to 22.33)
Central venous catheter (1 to 3)	272 (77.3)	81 (92)	191 (72.3)	< 0.001	4.42 (1.95 to 10.02)
Surgery (-7 to 0)	40 (11.4)	11 (12.5)	29 (11)	0.700	1.16 (0.55 to 2.43)
Surgery (-7 to 3)	224 (63.6)	62 (70.5)	162 (61.4)	0.159	1.50 (0.89 to 2.53)
Abdominal surgery (-7 to 3)	92 (26.1)	40 (45.5)	52 (19.7)	< 0.001	3.40 (2.02 to 5.70)
Immunosuppressants (-7 to 0)	38 (10.8)	15 (17)	23 (8.7)	0.045	2.15 (1.07 to 4.34)
Pancreatitis (-7 to 3)	6 (1.7)	2 (2.3)	4 (1.5)	0.642	1.51 (0.27 to 8.40)
TPN (1 to 3)	69 (19.6)	33 (37.5)	36 (13.6)	< 0.001	3.80 (2.18 to 6.63)
Dialysis (1 to 3)	32 (9.1)	10 (11.4)	22 (8.3)	0.396	1.41 (0.64 to 3.11)
Systemic corticosteroids (-7 to 3)	134 (38.1)	41 (46.6)	93 (35.2)	0.076	1.60 (0.98 to 2.62)
Diabetes	101 (28.7)	26 (29.5)	75 (28.4)	0.892	1.06 (0.62 to 1.80)
Mechanical ventilation (-7 to 3)	210 (59.7)	56 (63.6)	154 (58.3)	0.413	1.23 (0.75 to 2.03)
Mean APACHE II score, day 1 (± SD)	15.9 (9.5)	17.0 (8.8)	15.5 (9.7)	0.195	1.03 (0.99 to 1.06)
Mean pre-ICU LOS, days (± SD)	1.7 (0.24)	3 (7.3)	1.3 (3.0)	0.036	1.08 (1.01 to 1.14)

OR, odds ratio; CI, confidence interval; TPN, total parenteral nutrition; APACHE, Acute Physiology and Chronic Health Evaluation; LOS, length of stay.

### Outcomes

The original publications of the prediction rules did not provide specific equation variables for each rule. Therefore, on the basis of multivariate analysis of each risk factor, we derived equations for each prediction rule (Table 3). Regardless of the statistical significance of the individual risk factors in our patient population (Table 2), the Paphitou and Ostrosky-Zeichner rules were applied as is to our study population to calculate the sensitivity, specificity, PPV, NPV and AUC ROC for our institution (Table 4). Sensitivity and specificity ranged from approximately 41% to 74% and 61% to 81%, respectively. The PPV was less than 5% for all rules except Paphitou 2, at 5.4%, and the NPV was greater than 98% for all rules. The NMC rule demonstrated a sensitivity of 84.1%, specificity of 60.2%, PPV of 4.7%, NPV of 99.4% and AUC ROC of 0.770 (Table 4). The rule breakpoint values are shown in Table 4. A significant increase in in-hospital mortality was seen in patients who developed invasive candidiasis compared to controls (29.5% vs. 15.2%, odds ratio 2.33, 95% confidence interval, 1.3 to 4.16; *P *= 0.004). Cases, as compared to matched controls, had significantly longer mean total overall hospital stay (45.3 days vs. 17.1 days; *P *< 0.001) and higher mean total costs ($151,940.84 vs. $53,355.56; *P *< 0.001).

**Table 3:** Prediction rule equations^a^

Rule	Equation
Paphitou 1	(0.092 × diabetes) + (1.445 × TPN D-7 to D0) + (0.46 × HD D1 to D3)
Paphitou 2	(0.065 × diabetes) + (1.352 × TPN D-7 to D0) + (0.436 × HD D1 to D3) + (0.523 × BSAbx D-7 to D3)
Ostrosky 1	(1.465 × BSAbx D1 to D3) + (-0.188 × surgery D-7 to D0) + (0.769 × immunosuppression D-7 to D0) + (1.41 × TPN D1 to D3) + (0.524 × HD1 to HD3) + (0.342 × steroid D-7 to D3)
Ostrosky 2	(1.116 × CVC D1 to D3) + (-0.183 × surgery D-7 to D0) + (0.782 × immunosuppression D-7 to D0) + (1.408 × TPN D1 to D3) + (0.468 × HD D1 to D3) + (0.377 × steroid D-7 to D3)
NMC	(1.537 × BSAbx D1 to D3) + (0.873 × CVC D1 to D3) + (0.922 × TPN D1 to D3) + (0.402 × steroid D-7 to D3) + (0.879 × abdominal surgery) + (0.039 × pre-ICU LOS)

^a^TPN, total parenteral nutrition; HD, hemodialysis; BSAbx, broad-spectrum antibiotics; CVC, central venous catheter; NMC, Nebraska Medical Center; LOS, length of stay.

**Table 4:** Comparison of prediction rules^a^

Rule	Sensitivity	Specificity	PPV	NPV	AUC ROC	Rule break point
Paphitou 1	0.409	0.811	0.048	0.983	0.649	0.506
Paphitou 2	0.456	0.811	0.054	0.984	0.692	0.556
Ostrosky 1	0.659	0.640	0.041	0.988	0.698	1.68
Ostrosky 2	0.739	0.606	0.042	0.990	0.705	1.14
NMC	0.841	0.602	0.047	0.994	0.770	2.45

^a^PPV, positive predictive value; NPV, negative predictive value; AUC ROC, area under the receiver operating characteristic curve; NMC, Nebraska Medical Center.

## Discussion

As shown in our study, invasive candidiasis is significantly associated with increased mortality, longer overall hospital stay and higher costs. Due to the appreciable morbidity, mortality and costs associated with invasive candidiasis and the implications of delayed appropriate antifungal therapy, the use of clinical prediction rules to help determine in which high-risk patients prophylactic antifungal therapy may be beneficial is of interest [2,4-7]. This study served as an external validation and comparison of two previously published clinical prediction rules for invasive candidiasis [15,16]. The variables utilized in this study were applied exactly as published in the original rules. In our patient population, several risk factors used in the Paphitou and Ostrosky-Zeichner rules, including surgery, pancreatitis, hemodialysis and diabetes, were not shown to have statistically significant correlations to invasive candidiasis. Conversely, abdominal surgery and pre-ICU length of stay, which are risk factors that were not used in the Paphitou and Ostrosky-Zeichner rules, were found to be significantly associated with invasive candidiasis. These factors were used in combination with other significant risk factors, such as the use of broad-spectrum antibiotics, presence of a CVC and use of TPN and systemic corticosteroids to develop a clinical prediction rule for our patient population.

The Paphitou and Ostrosky-Zeichner prediction rules performed similarly. Paphitou rule 1, involving the fewest risk factors, demonstrated the lowest sensitivity but the highest specificity. As the rules utilized more risk factors, the sensitivity increased with a corresponding drop in specificity. The NMC rule demonstrated a higher sensitivity than and similar specificity to the Ostrosky-Zeichner rule with the same number of risk factors used in the rule (Tables 3 and 4). Depending on the rule, only 4.1% to 5.4% of patients meeting rule criteria developed invasive candidiasis. This low PPV suggests a limited role for the prediction rules in identifying patients most likely to benefit from antifungal prophylaxis in the ICU. Because of a relatively low overall incidence of invasive candidiasis, all of the prediction rules had a low PPV, which means that if the NMC rule had been applied to our study population, the percentage of ICU patients who would have received antifungal prophylaxis would have been 4.7%. In contrast, the rules demonstrated a high NPV. The NPVs indicate that fewer than 2% (fewer than 1% under the NMC rule) of patients not meeting rule criteria developed invasive candidiasis. This suggests that when the rule equations are applied to a patient, if the resultant value is below the rule breakpoint (Table 4), the patient will not likely develop invasive candidiasis (for example, a probability of 99% using the NMC rule) and therefore will not benefit from antifungal prophylaxis.

Paphitou's rule was developed in 2005 on the basis of a study of 327 patients in a surgical ICU [15]. The overall incidence of invasive candidiasis in the Paphitou study was 7.1%, which is higher than the observed overall incidence of 2.3% in our study. However, our study was not conducted exclusively in a surgical ICU population. Direct comparison of the sensitivity, specificity, PPV and NPV is not possible, because these values were not reported in the Paphitou study.

In 2007, Ostrosky-Zeichner and colleagues [16] expanded on the results of the Paphitou study to include a broader range of risk factors in both medical and surgical ICU patients in a multicenter study of nearly 3,000 patients. In contrast to the Paphitou study, the overall incidence of invasive candidiasis among the Ostrosky-Zeichner population, at 3%, was more similar to that of our patient population. Likewise, the Ostrosky-Zeichner patient population was more comparable to ours in that medical and surgical ICU patients were included. Sensitivity and specificity were 34% and 90%, respectively, and the PPV and NPV were 1% and 97%, respectively. In comparison, our study demonstrated higher sensitivity, PPV and NPV, but lower specificity.

More recently, Ostrosky-Zeichner and colleagues [17] further refined their rule in a retrospective study of 597 ICU patients by including the combination of mechanical ventilation for at least 48 hours and the presence of a CVC and broad-spectrum antibiotic use on D1 to D3 plus an additional minor risk factor and found that this changed the sensitivity, specificity, PPV and NPV to 50%, 83%, 10% and 97%, respectively. The investigators also applied their original rule to this patient population and found a sensitivity and specificity of 27% and 93%, respectively, and a PPV and NPV of 13% and 97%, respectively. In comparison, our study demonstrated higher sensitivity and NPV, but lower specificity and PPV. The overall incidence of invasive candidiasis in the Ostrosky-Zeichner population was 3.7%. Notably, 80% of patients in the Ostrosky-Zeichner study were surgical ICU patients, and 78% of them were mechanically ventilated in comparison to approximately 60% who were mechanically ventilated in our study. Moreover, mechanical ventilation was not found to be significantly different among cases and controls in our study population (Table 2). Because our study was completed before the new rule was published, comparison of the sensitivity, specificity, PPV and NPV of this rule in our patient population was not possible.

Notably, a large number of solid organ and hematopoietic stem cell transplants are performed at our institution. Because the specific equations used in the Paphitou and Ostrosky-Zeichner studies were not reported, we had to derive our own equations for their rules. Our patient population's characteristics may have caused various factors, such as use of immunosuppressants, systemic corticosteroids and broad-spectrum antibiotics, to be weighted differently in the equations for the rules. Consequently, these factors might have changed the performance of the rules.

The AUC ROC curve is a measure of the discriminative power of the rule. A value of 1 would be equivalent to perfect discriminative power; that is, the higher and closer a value is to unity, the better the rule discriminates patients with invasive candidiasis from patients without invasive candidiasis. Paphitou rule 1, incorporating the fewest risk factors, achieved the lowest AUC ROC curve and therefore the lowest discriminative power. Our institution-specific rule had the highest AUC ROC curve at 0.770, indicating the best discriminative power among the various rules.

Playford *et al. *[18] recently evaluated three clinical prediction rules in a prospective cohort of 615 Australian ICU patients. The rules evaluated included the 2007 and 2011 Ostrosky-Zeichner rules plus an additional rule that included *Candida *colonization [16,17,21]. Fungal surveillance cultures of the throat, rectum and/or perineum and urine were performed 72 hours after ICU admission and twice weekly thereafter, enabling the investigators to add *Candida *colonization as a factor to the two Ostrosky-Zeichner rules. The overall incidence of invasive candidiasis in their study was 2.4%, which is similar to the incidence in our study. However, mechanical ventilation, CVC use and surgical procedures were more common and systemic corticosteroid and immunosuppressant use were less common in their study than in our patient population. Their study demonstrated that the specificities and PPVs of the two Ostrosky-Zeichner rules were improved by the addition of colonization to the rule, although this resulted in lower sensitivity.

Our study has several limitations. First, the study was a retrospective, single-center study. Additionally, our institution has a large population of solid organ transplant and hematopoietic stem cell transplant patients. Thus, the results of our study may not be generalizable to those conducted at other institutions. Data from our study as well as that of Playford *et al. *[18] suggest that clinical prediction rules can be applied only to patient populations other than the derivation population if the potential for case-mix variability is taken into consideration. The application of the rules was limited to ICU patients, as this was the original population for which the rules were developed. The development and application of a prediction rule for use in the non-ICU patient population remains an unaddressed issue. Also, previous studies supporting the effectiveness of antifungal prophylaxis were conducted in populations that had an incidence of invasive candidiasis over 10% among the control populations [9-11]. In contrast, our study, along with those in which the Ostrosky-Zeichner and Paphitou rules were developed [15-17], occurred in populations with lower infection rates. In this setting, it may be more appropriate to focus attention on early empiric antifungal therapy, although early empiric therapy with fluconazole was not shown to be beneficial in an ICU population with an incidence of invasive candidiasis of 9% in the control population [22]. Last, clinical prediction rules utilizing colonization with *Candida *spp. as a risk factor [21,23] could not be evaluated in our study, because routine surveillance cultures to detect such colonization are not performed at our institution. While colonization has been shown to be a risk factor for invasive candidiasis [24], the predictive value of colonization for invasive candidiasis is variable [21,23,25].

## Conclusions

Invasive candidiasis is a serious and devastating infection leading to a significant clinical and economic burden on patients and healthcare systems. The best method for identifying patients who are likely to benefit from antifungal prophylaxis remains unclear. Clinical prediction rules incorporating numerous risk factors have been developed and externally applied. A new clinical prediction rule, the NMC rule, using factors that are easily obtained, was developed on the basis of our experience in our patient population and compared favorably to the previously published rules. We intend to validate this rule in a prospective study at our institution. Because of the high NPV, the rules are likely best applied to identify patients who would least likely benefit from antifungal prophylaxis rather than to identify patients who should receive such therapy. Future studies should investigate the prospective application of these clinical prediction rules in an attempt to prevent unnecessary antifungal therapy, optimize care for patients and facilitate the design of forthcoming antifungal clinical trials.

## Key messages

• The prevalence of nosocomial invasive candidiasis has increased and is associated with increased mortality, longer hospital stay and higher costs. Further, delays in appropriate therapy have been associated with increased mortality due to invasive candidiasis.

• Universal antifungal prophylaxis is not clinically or fiscally responsible, as many patients are at low risk for developing infection, and inappropriate use of antifungals can promote resistance and increase costs. Therefore, a prediction rule for identifying patients most likely to benefit from prophylaxis would be advantageous.

• This study serves as an external validation and comparison of two clinical prediction rules for invasive candidiasis previously published by Paphitou *et al. *[15] and Ostrosky-Zeichner *et al. *[16]. A new clinical prediction rule, the NMC rule, using factors that are easily obtained, was developed on the basis of our experience in our patient population and compared favorably to the previously published rules.

• Due to the high NPVs, the rules are likely best applied to identify patients who would be least likely to benefit from antifungal prophylaxis rather than to identify patients who should receive such therapy.

• Future studies should investigate the prospective application of these clinical prediction rules in an attempt to prevent unnecessary antifungal therapy, optimize care for patients and facilitate the design of forthcoming antifungal clinical trials.

## Abbreviations

AUC ROC: area under the receiver operating characteristic curve; CVC: central venous catheter; NPV: negative predictive value; PPV: positive predictive value; TPN: total parenteral nutrition.

## Competing interests

EDH is currently an employee of Cubist Pharmaceuticals; is a shareholder in Cubist Pharmaceuticals; has been a consultant for Ortho-McNeil Pharmaceuticals, Cubist Pharmaceuticals and Forest Laboratories; and has received research funding from Pfizer and TheraDoc. MER has received research funding from Cubist Pharmaceuticals, Sanofi-Pasteur, Cardinal Health Foundation, 3 M and Molynlcke. AGF has received research funding from Merck and Pfizer. MKZ, MM and ACK have no conflicts of interest to declare.

## Authors' contributions

EDH contributed to the study concept and design, the execution of the study and manuscript preparation. MKZ and MM contributed to data acquisition and manuscript preparation. MER and AGF contributed to the study design and manuscript preparation. ACK contributed to the study design, statistical analysis and manuscript preparation. All authors read and approved the final manuscript.

## References

[B1] Fridkin SK Jarvis WR Epidemiology of nosocomial fungal infections Clin Microbiol Rev 1996 9 499 511 8894349 10.1128/cmr.9.4.499 PMC172907

[B2] Wisplinghoff H Bischoff T Tallent SM Seifert H Wenzel RP Edmond MB Nosocomial bloodstream infections in US hospitals: analysis of 24,179 cases from a prospective nationwide surveillance study Clin Infect Dis 2004 39 309 317 10.1086/421946 15306996

[B3] Blumberg HM Jarvis WR Soucie JM Edwards JE Patterson JE Pfaller MA Rangel-Frausto MS Rinaldi MG Saiman L Wiblin RT Wenzel RP National Epidemiology of Mycoses Survey (NEMIS) Study Group Risk factors for candidal bloodstream infections in surgical intensive care unit patients: the NEMIS prospective multicenter study. The National Epidemiology of Mycosis Survey Clin Infect Dis 2001 33 177 186 10.1086/321811 11418877

[B4] Rentz AM Halpern MT Bowden R The impact of candidemia on length of hospital stay, outcome, and overall cost of illness Clin Infect Dis 1998 27 781 788 10.1086/514955 9798034

[B5] Garey KW Rege M Pai MP Mingo DE Suda KJ Turpin RS Bearden DT Time to initiation of fluconazole therapy impacts mortality in patients with candidemia: a multi-institutional study Clin Infect Dis 2006 43 25 31 10.1086/504810 16758414

[B6] Morrell M Fraser VJ Kollef MH Delaying the empiric treatment of *Candida *bloodstream infection until positive blood culture results are obtained: a potential risk factor for hospital mortality Antimicrob Agents Chemother 2005 49 3640 3645 10.1128/AAC.49.9.3640-3645.2005 16127033 PMC1195428

[B7] Nolla-Salas J Sitges-Serra A León-Gil C Martínez-González J León-Regidor MA Ibáñez-Lucia P Torres-Rodríguez JM Candidemia in non-neutropenic critically ill patients: analysis of prognostic factors and assessment of systemic antifungal therapy. Study Group of Fungal Infection in the ICU Intensive Care Med 1997 23 23 30 10.1007/s001340050286 9037636

[B8] Cruciani M de Lalla F Mengoli C Prophylaxis of *Candida *infections in adult trauma and surgical intensive care patients: a systematic review and meta-analysis Intensive Care Med 2005 31 1479 1487 10.1007/s00134-005-2794-y 16172847

[B9] Eggimann P Francioli P Bille J Schneider R Wu MM Chapuis G Chiolero R Pannatier A Schilling J Geroulanos S Glauser MP Calandra T Fluconazole prophylaxis prevents intra-abdominal candidiasis in high-risk surgical patients Crit Care Med 1999 27 1066 1072 10.1097/00003246-199906000-00019 10397206

[B10] Garbino J Lew DP Romand JA Hugonnet S Auckenthaler R Pittet D Prevention of severe *Candida *infections in nonneutropenic, high-risk, critically ill patients: a randomized, double-blind, placebo-controlled trial in patients treated by selective digestive decontamination Intensive Care Med 2002 28 1708 1717 10.1007/s00134-002-1540-y 12447512

[B11] Pelz RK Hendrix CW Swoboda SM Diener-West M Merz WG Hammond J Lipsett PA Double-blind placebo-controlled trial of fluconazole to prevent candidal infections in critically ill surgical patients Ann Surg 2001 233 542 548 10.1097/00000658-200104000-00010 11303137 PMC1421284

[B12] Savino JA Agarwal N Wry P Policastro A Cerabona T Austria L Routine prophylactic antifungal agents (clotrimazole, ketoconazole, and nystatin) in nontransplant/nonburned critically ill surgical and trauma patients J Trauma 1994 36 20 26 10.1097/00005373-199401000-00004 8295245

[B13] Playford EG Webster AC Sorrell TC Craig JC Antifungal agents for preventing fungal infections in non-neutropenic critically ill and surgical patients: systematic review and meta-analysis of randomized clinical trials J Antimicrob Chemother 2006 57 628 638 10.1093/jac/dki491 16459344

[B14] Shorr AF Chung K Jackson WL Waterman PE Kollef MH Fluconazole prophylaxis in critically ill surgical patients: a meta-analysis Crit Care Med 2005 33 1928 1936 10.1097/01.CCM.0000178352.14703.49 16148461

[B15] Paphitou NI Ostrosky-Zeichner L Rex JH Rules for identifying patients at increased risk for candidal infections in the surgical intensive care unit: approach to developing practical criteria for systematic use in antifungal prophylaxis trials Med Mycol 2005 43 235 243 10.1080/13693780410001731619 16010850

[B16] Ostrosky-Zeichner L Sable C Sobel J Alexander BD Donowitz G Kan V Kauffman CA Kett D Larsen RA Morrison V Nucci M Pappas PG Bradley ME Major S Zimmer L Wallace D Dismukes WE Rex JH Multicenter retrospective development and validation of a clinical prediction rule for nosocomial invasive candidiasis in the intensive care setting Eur J Clin Microbiol Infect Dis 2007 26 271 276 10.1007/s10096-007-0270-z 17333081

[B17] Ostrosky-Zeichner L Pappas PG Shoham S Reboli A Barron MA Sims C Wood C Sobel JD Improvement of a clinical prediction rule for clinical trials on prophylaxis for invasive candidiasis in the intensive care unit Mycoses 2011 54 46 51 10.1111/j.1439-0507.2009.01756.x 19627509

[B18] Playford EG Lipman J Kabir M McBryde ES Nimmo GR Lau A Sorrell TC Assessment of clinical risk predictive rules for invasive candidiasis in a prospective multicentre cohort of ICU patients Intensive Care Med 2009 35 2141 2145 10.1007/s00134-009-1619-9 19756510

[B19] De Pauw B Walsh TJ Donnelly JP Stevens DA Edwards JE Calandra T Pappas PG Maertens J Lortholary O Kauffman CA Denning DW Patterson TF Maschmeyer G Bille J Dismukes WE Herbrecht R Hope WW Kibbler CC Kullberg BJ Marr KA Muñoz P Odds FC Perfect JR Restrepo A Ruhnke M Segal BH Sobel JD Sorrell TC Viscoli C Wingard JR Zaoutis T Bennett JE European Organization for Research and Treatment of Cancer/Invasive Fungal Infections Cooperative Group; National Institute of Allergy and Infectious Diseases Mycoses Study Group (EORTC/MSG) Consensus Group Revised definitions of invasive fungal disease from the European Organization for Research and Treatment of Cancer/Invasive Fungal Infections Cooperative Group and the National Institute of Allergy and Infectious Diseases Mycoses Study Group (EORTC/MSG) Consensus Group Clin Infect Dis 2008 46 1813 1821 10.1086/588660 18462102 PMC2671227

[B20] Rothman KJ Greenland S Lash TL Modern Epidemiology 2008 3 Philadelphia Lippincott Williams & Wilkins

[B21] Pittet D Monod M Suter PM Frenk E Auckenthaler R *Candida *colonization and subsequent infections in critically ill surgical patients Ann Surg 1994 220 751 758 10.1097/00000658-199412000-00008 7986142 PMC1234477

[B22] Schuster MG Edwards JE Jr Sobel JD Darouiche RO Karchmer AW Hadley S Slotman G Panzer H Biswas P Rex JH Empirical fluconazole versus placebo for intensive care unit patients: a randomized trial Ann Intern Med 2008 149 83 90 18626047 10.7326/0003-4819-149-2-200807150-00004

[B23] León C Ruiz-Santana S Saavedra P Almirante B Nolla-Salas J Alvarez-Lerma F Garnacho-Montero J León MA EPCAN Study Group A bedside scoring system ("*Candida *score") for early antifungal treatment in nonneutropenic critically ill patients with *Candida *colonization Crit Care Med 2006 34 730 737 10.1097/01.CCM.0000202208.37364.7D 16505659

[B24] Wey SB Mori M Pfaller MA Woolson RF Wenzel RP Risk factors for hospital-acquired candidemia: a matched case-control study Arch Intern Med 1989 149 2349 2353 10.1001/archinte.149.10.2349 2802900

[B25] Pelz RK Lipsett PA Swoboda SM Diener-West M Hammond JM Hendrix CW The diagnostic value of fungal surveillance cultures in critically ill patients Surg Infect (Larchmt) 2000 1 273 281 10.1089/109629600750067200 12594883

